# Reading Faces: Differential Lateral Gaze Bias in Processing Canine and Human Facial Expressions in Dogs and 4-Year-Old Children

**DOI:** 10.1371/journal.pone.0036076

**Published:** 2012-04-27

**Authors:** Anaïs Racca, Kun Guo, Kerstin Meints, Daniel S. Mills

**Affiliations:** 1 School of Psychology, University of Lincoln, Lincoln, United Kingdom; 2 School of Life Sciences, University of Lincoln, Lincoln, United Kingdom; Ghent University, Belgium

## Abstract

Sensitivity to the emotions of others provides clear biological advantages. However, in the case of heterospecific relationships, such as that existing between dogs and humans, there are additional challenges since some elements of the expression of emotions are species-specific. Given that faces provide important visual cues for communicating emotional state in both humans and dogs, and that processing of emotions is subject to brain lateralisation, we investigated lateral gaze bias in adult dogs when presented with pictures of expressive human and dog faces. Our analysis revealed clear differences in laterality of eye movements in dogs towards conspecific faces according to the emotional valence of the expressions. Differences were also found towards human faces, but to a lesser extent. For comparative purpose, a similar experiment was also run with 4-year-old children and it was observed that they showed differential processing of facial expressions compared to dogs, suggesting a species-dependent engagement of the right or left hemisphere in processing emotions.

## Introduction

Being sensitive to other's emotions provides a clear biological advantage in the form of harm avoidance, access to resources or facilitation of group cohesion. In humans, facial expressions are probably the main way of communicating emotions [1]. In order to understand the phylogenetic origins of facial communication of emotions, researchers have concentrated their effort on the study of primate species, offering detailed reports regarding the production and processing of facial movements within this taxon [2]–[9]. As facial communication is largely suggested to be an adaptation to a complex social life [3], [9], a specialized system dedicated to facial communication should also be found in other social mammalian species. In this respect, social canids are known to present a large range of facial expressions [9], [10]; however, the way they process them has not been empirically investigated yet.

Since Darwin [11] first initiated the study of comparative expressions of emotions across species, studies revealed that some aspects of facial expressions and their function seems to be preserved across diverse animal taxa, suggesting a possible similar evolutionary root for them; while others appear to be species-specific, highlighting that animal species also evolved facial signals dependent on their specific social and ecological needs [12], [13]. Thus, although an accurate processing of other species' facial emotions could be advantageous in inter-specific co-habitation, such as occurs between dogs and humans, it could be challenged by the species-specificity of some signals, which would prevent animals from relying on simple homologies, particularly between distant animal taxa.

Dogs being long domesticated animals (12,000–33,000 [14], [15] years ago) and occupying a close social anthropogenic niche since this time would benefit considerably from an appropriate reading of humans' facial communication. In the last twenty years, numerous studies have highlighted dogs' abilities to read some human communicative visual signals such as pointing, gazing or nodding in the direction of a target [16], [17]. They also present a certain sensitivity to human faces and can even pick up some important information from them. For instance, dogs attend to human faces to assess their attentional state [18]–[20] and can follow human eye/head direction to find hidden food [17], [21]. They can discriminate between 2D pictures of unfamiliar human faces [22] and they present a decrease in attention towards their owner if the latter's head is not visible [23]. Dogs may even have an internal representation of their owner's face [24]. Few studies experimentally assessed dogs' sensitivity to emotional communication by humans. It has been found for instance that dogs can discriminate between agonistic and affiliative human body postures [25], [26] and between some emotionally different tones of voice [27], [28]. Regarding facial expressions, studies showed that dogs react differently to actors performing a range of emotional facial expressions (e.g. anger, fear) compared to neutral ones [29] and can discriminate between smiling and blank faces using photographs [30]. Although these studies indicate that dogs can pick up some emotional information from human faces, the way they process these and notably in comparison to the way they process facial expressions from their own species, is still unknown.

Visual processing of emotions is known to be subject to brain lateralisation in both human and non-human animals [31], [32]. Two main theories have been proposed regarding the type of lateralisation involved. The *Right Hemisphere Model* suggests that the right hemisphere regulates emotional processes, regardless of their valence [33] and the *Valence Model* states that the right hemisphere is mainly involved regarding negative emotions and the left hemisphere is mainly involved regarding positive emotions [34]. Both theories have been supported by empirical studies using behavior measurements (i.e. contralateral visual field advantage, lateral gaze bias), clinical investigations (i.e. patients with lateralised brain lesions), electrophysiological and neuroimaging approaches [35]. Regarding domestic dogs, researchers studied asymmetrical head-orienting responses towards emotional visual stimuli and found a right hemispherical dominance (head turning preferentially towards the left) to threatening stimuli (e.g. the silhouette of a snake) [36]. Right hemispherical dominance was also observed in dogs towards intense emotional stimuli through both acoustic (e.g. thunderstorm [37]) and olfactory (e.g. veterinary sweat [38]) sensory channels. Asymmetrical behaviours associated with hemispherical lateralisation towards emotional stimuli in dogs have also been found regarding tail-wagging [39], where the dogs preferentially wagged their tail to the left (right hemisphere) while presented with emotionally negative stimuli (i.e. a dominant dog) and to the right (left hemisphere) with positive stimuli (i.e. the dog's owner).

In order to assess dogs' sensitivity to both dog and human facial communication, we examined their visual lateralisation towards the facial expressions of both species associated with different emotional valence [negative (threatening), neutral and positive (friendly)]. In species with frontal eyes, such as primates or canids, continuous binocular vision is used and each visual field relays mainly to the contralateral hemisphere. Therefore, a preference for one or the other visual field, revealed from lateral eye movements (i.e. ‘gaze bias’), is associated with the engagement of the opposite brain hemisphere. In a previous study [40] it was found that domestic dogs display a left gaze bias when viewing human faces with neutral expressions, interpreted as a right hemispherical dominance to process these, as is the case in humans and other primates [40], [41]. Interestingly dogs did not present such a bias towards faces of their own species. In the case that dogs are responsive to human and/or dog facial emotions, we would expect them to display variations in their gaze bias between emotionally expressive faces and neutral ones. The direction (left, right) of these variations should reflect the dominant use of one or the other brain hemisphere to process emotional facial expressions, in line with either the *Right Hemisphere Model* or the *Valence Model* mentioned earlier.

Dogs' developmental environment is comparable to that of children and similarities between dogs and infants socio-behavioural traits have been accumulating over the last decade [42]. Depending on the type of cognitive skill tested, the performance of dogs has been aligned to different stages of human development [43], [44], [45]. In humans the processing and understanding of the facial expression of emotions develops throughout entire childhood, reaching adult performances levels at around 10 years of age or even later [46]. Some rudimentary abilities are observed in young infants and even newborns, such as the ability to differentiate some common facial expressions [47], but the ability to correctly interpret the meaning of facial signals appears within the second year of life, as revealed when using simple forced choice between 2 pictures [48]. However, it is only by the age of 4 that children can categorize the most fundamental facial emotions correctly in a total free choice paradigm (i.e. happy, angry, sad, surprised) [49]. Studies on hemispherical differences in brain activity regarding the processing of facial expressions through development present a similar discrepancy between the *Right Hemisphere* and the *Valence* models as those found in studies with adult participants [50], [51].

In this study, for the purpose of investigating how similar dogs' processing of facial expressions is to humans', we systematically compared dogs' behavioural responses to that of 4-year-old children, since this is the age by which fundamental facial expressions appear to be correctly interpreted by children.

## Methods

### Participants

Ethical approval was granted by the University of Lincoln (UK) for this study and procedures complied with the ethical guidance of the International Society for Applied Ethology regarding animal subjects and the British Psychological Society Ethical guidance regarding children participants. Children received a debriefing following the study regarding dog communication and emotional state as well as safe behaviour when interacting with dogs. The “Blue Dog” CD, an interactive computer animation aiming to educate parents and children about safe interaction with dogs [52] was also offered to all children and parents.

### Dogs

Thirty-seven healthy adult pet dogs, all well-socialised with both humans and other dogs, were recruited for this experiment by e-mail announcements among the staff of the University of Lincoln.

Twenty-two of them successfully completed the study. The main reasons for failure to complete related to a lack of attention and/or restlessness (5 dogs), distress (4 dogs) or deviation from the instructions by the accompanying person (6 dogs). One of the 22 dogs who completed the procedure was excluded from the data analysis for producing scores above 2.5 standard deviations from the mean, and so was rejected as an outlier. The final sample contained 21 dogs (10 males and 11 females; 5 Border Collies, 4 Labradors, 2 Lurchers, 1 German Shepherd , 1 miniature Dachshund, 1 Grey Hound, 1 Jack Russell and 6 cross-breeds), aged between 1 and 9 years of age with an average of 4.62±0.61 years (mean±SE).

#### Children

Twenty-five healthy, right handed, 4-year-old children were recruited for this study through leaflets to the parents for participation in child development studies delivered to nurseries, playgroups, etc. Nineteen of them successfully completed the experiment. Among the 6 children excluded from the analysis, 3 of them were the result of parental withdrawal, 2 of them showed a lack of attention and 1 of them was excluded due to equipment failure. Two further children were excluded as outliers (scores above 2.5 standard deviations from the mean). The final sample contained 17 children, 5 boys and 12 girls (age ranging from 3.89 to 4.11 years old with the mean of 4.01±0.01 years old).

### Visual stimuli

**Figure 1:**
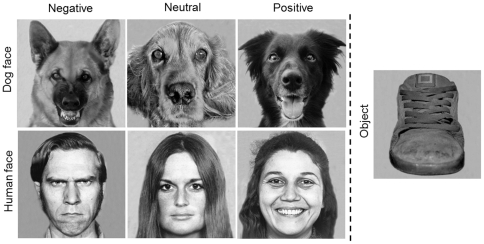
Example of the used stimuli.

Twenty-eight grey-scale pictures were used in this study: 12 pictures of dog faces, 12 pictures of human faces and 4 pictures of objects. Both dog and human pictures were divided into 3 categories (with 4 pictures for each category) corresponding to the valence of the facial expression displayed: negative (threatening), neutral and positive (friendly). An example of the pictures used can be seen in Figure 1.

The pictures of dog faces were taken by an experimenter except for 2 pictures used from the internet. The pictures were taken in different situations associated with different emotional states and eliciting distinctive facial expressions. Pictures of negative facial expressions were chosen from dogs displaying typically threatening aggressive facial signals: bared-teeth, wrinkled muzzle, erect and forward pointing ears. One of these pictures was obtained from a police dog, trained to display such behaviour and one came from a dog in a kennel presenting aggressive behaviour towards strangers; two other pictures of threatening dogs were obtained from the internet. For the positive facial expressions of dogs, pictures were taken when presenting dogs with food (just above the camera) and talking to them using ‘doggerel’ (similar to ‘baby speech’ but directed to dogs) [53]. The typical facial reaction was a relaxed face with an open mouth, the tongue out and erect ears. Regarding the neutral situation, the photographer waited for the dog not to be involved in any activity and to ignore her, appearing to be relaxed and present no obvious facial muscle tension. Human faces were selected from the “Pictures of Facial Affect” by Ekman and Friesen [54]. This validated and widely used database consists of a series of pictures from Caucasian actors and actresses trained to display facial expressions. The facial expressions chosen were ‘angry’, ‘neutral’ and ‘happy’ (2 men and 2 women for each expression). Both human and dog pictures were taken from a front view and presented with a straight gaze and no obvious lateralised facial marks. All faces presented in the pictures were unknown to the participants. The object pictures consisted of 4 different objects, chosen to be quasi-symmetrical items (such as faces) and covering a large horizontal space in order to elicit lateral eye movements. The category of object presented was familiar to the tested dogs (a shoe, a flower, a house, a tree) but the specific items presented were novel.

The pictures were processed in Adobe Photoshop 7.0 to ensure similar size of stimuli (600×600 pixels resolution) and uniform background. The contrast and brightness of the pictures was also visually adjusted to appear similar between all pictures. Additionally, to control for an effect of the pictures' proprieties on the participants' lateral eye movements, the left and the right side of the pictures were compared according to 2 main objective photometric measures: luminance (cd/m^2^) and contrast (percentage of ‘grey pixels’ within the image, the ‘grey pixels’ corresponding to 2/3 of the distribution of gray scale from black to white). Both measures were averaged for each stimulus category and there were no significant differences between the right and the left side of the pictures (2-tailed paired *t*-tests, *p*>0.05).

### Experimental protocol

**Figure 2:**
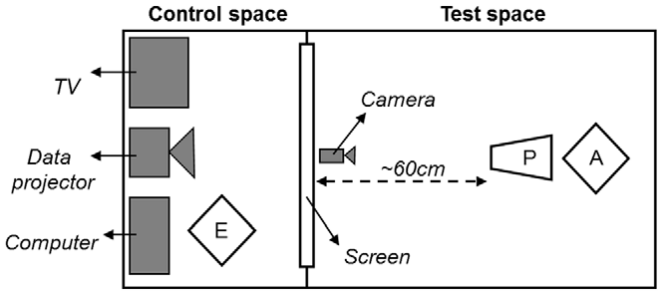
Schematic apparatus. E: experimenter, P: participant, A: accompanying person.

The pet dogs were tested in the Animal Cognition Laboratory of the Department of Biological Sciences and the children were tested at the Lincoln Infant Lab within the School of Psychology of the University of Lincoln (UK). A similar apparatus was used for both dogs and children and is schematised in Figure 2.

The dogs were first familiarised with a quiet test room for a few minutes. Either the dog owner or an experimenter (previously familiarised to the dog) accompanied the dogs during the entire test. The dog was sat about 60cm in front of a projection screen (giving the pictures a visual angle of 57×57°) and the carer stood behind the dog and put her/his hands on the dogs' shoulders. The accompanying person did not interfere with the dog during the picture presentation or force it to watch the screen. A CCTV camera (SONY SSC-M388CE, resolution: 380 horizontal lines) placed in front of the dog, at the bottom of the screen, was used to monitor and record the dog's eye movements. An experimenter, in a control room (behind the screen, not visible to the dogs), was in charge of attracting the dog's attention and presenting visual stimuli on the screen. A TV screen, linked to the video camera, allowed live monitoring of the dog's face during the study. In order to attract the dog's attention towards the middle of the screen, the experimenter first used a sound stimulus (e.g. a call to the dog, tap on the screen, etc.), then projected a small fixation point (FP) which expanded and contracted in the centre of the screen (ranging between 2.8 and 6.6°). Once the dog's gaze was oriented towards the FP, that is, looking straight above the camera, a visual stimulus was presented for 5 seconds and the dog could passively look at the picture for as long as it wanted. The pictures were presented in a random order for each dog and the presentation of each picture was preceded with the same procedure. The dogs were allowed breaks when needed (i.e. an attention drop).

The procedure with children was similar to the one used with dogs. They sat on the knees of one of their parents and the latter was asked to not interfere with the child during the picture presentation. To attract the children' s attention to the middle of the screen a FP was projected accompanied by a female auditory instruction of “look” delivered through a loudspeaker positioned centrally above the displayed picture. Children could take breaks when needed.

As several studies have shown that humans do not express their emotions in a symmetrical way between the left and the right hemiface [55] we needed to control for a direct effect of these potential asymmetries in our stimuli on the behaviour of the tested subjects. Thus, 16 of the 21 dogs and 15 of the 17 children (5 dogs and 2 children were not available for a second session) were also tested on a different session following an identical protocol in which they were presented with the same stimuli but as a mirror image (i.e. the left side of the picture was then presented on the right and *vice-versa*). The order of presentation of the ‘standard’ and the ‘mirror’ sessions was balanced between subjects.

### Data analysis and statistics

The dogs' and children's eye movements were recorded and then digitised with a sampling frequency of 60 Hz. The videos were replayed off-line frame by frame and the gaze direction towards the screen was manually classified as ‘left’, ‘right’, ‘central’ and ‘out’ by a researcher, blind towards the picture presented. For further details of the coding see our previous study [22]. A trial was accepted if (1) the participant was looking at the centre of the screen when the picture was displayed and (2) if the participant presented at least one lateral eye movement towards the pictures during its presentation. All dogs tested successfully completed at least 75% of the trials (77%±3 in ‘standard sessions’ and 76%±2 in ‘mirror sessions’) and all children tested successfully completed at least 78% of the trials (90%±1 in ‘standard sessions’ and 93%±1 in ‘mirror sessions’). Cronbach's Alpha coefficient conducted on 10% of the data (‘standard session’) indicated good reliability between independent coders for both populations (dogs: 0.85; children: 0.81).

Data were first analysed within each group of participants separately (dogs and children) and were then analysed together in order to assess potential differences between both groups. Lateralisation in looking behaviour was assessed regarding two types of measures: *First look* and *Total look*.

### *First look*

This corresponds to the side of first fixation towards the pictures presented. For each subject and each image category presented, the lateral asymmetries of the *First look* were computed using the Laterality Index (LI): LI (First look) = (L−R/L+R) where L and R indicate, respectively, the number of left and right first fixations. Therefore, positive scores indicate a bias towards the left, negative scores indicate a bias towards the right and null scores indicates no bias. Friedman's tests were conducted for each face category (dog and human faces) to test for an effect of the emotional valence (negative, neutral, positive). Post-hoc comparisons were assessed using 2-tailed Wilcoxon Signed Rank tests. Significant bias towards the left or right side was estimated for each image category by comparing the data to chance level (0) using 2-tailed Wilcoxon Signed Rank tests.

#### Total look

This corresponds to the cumulative viewing time within a trial on the left and the right side of the pictures. For each subject and each image category presented, the *Total look* was converted to the Laterality Index (LI): LI (Total look) = (L−R/L+M+R) where L, M and R indicate, respectively, the time spent looking to the left, middle and right side of the pictures. Therefore, positive scores indicate a bias towards the left, negative scores indicate a bias towards the right and null scores indicate no bias.

Data distribution was checked for normality using a Shapiro-Wilk test (*p*>0.05) and equality of variance was assessed with Mauchly's sphericity test (*p*>0.05) for both dog and child participants. This allowed a 2-way analysis of variance (ANOVA) to be conducted on the LI (Total look) regarding the face pictures, considering the following factors: face category (dog, human) and emotional valence (negative, neutral, positive). *Post-hoc* tests (Fisher's protected LSD) were then conducted within separate ANOVAs in order to identify the origin of the effects. Significant variations from chance level (0), indicating lateralisation, were estimated using 2-tailed 1-sample *t*-tests for each image category (faces and objects).

#### Standard vs. Mirror sessions

To examine the effect of the session type (standard vs. mirror) regarding each group of participant, the scores obtained in both sessions were compared for each image category using 2-tailed Wilcoxon Signed Rank tests for LI (First look) and 2-tailed *t*-tests for LI (Total look).

#### Other measures

The total amount of time spent looking at each image category, the amount of interchanged fixations between the left and right side of the pictures as well as the latency of first detectable eye movements were also analysed regarding each group of participant. To assess for an effect of the type of pictures presented, Friedman tests were used as these measures were not normally distributed (Shapiro-Wilk test, *p*<0.05) for both dog and child participants.

#### Comparison between dogs and children

Given that a combined within- and between-subject analysis is possible only for parametric tests, the potential difference in the laterality of eye movement between dogs and 4-year-old children was estimated using only the *Total Look* measure. A 2-way mixed ANOVA was conducted with within-subject variables of face category (dog, human), emotional valence (negative, neutral, positive); and between-subject variable of participant type (dogs, children). The effect of face category and emotional valence being assessed independently for each type of participant in previous analysis, the focus of this ANOVA is to detect potential interactions between within-subject variables and participant type. Further separate 1-way ANOVAs were used to estimate the origin of these interactions.

Dogs and children were also compared regarding the amount of time spent looking at the images, the amount of interchanged fixations between the left and right side of the pictures as well as the latency of first detectable eye movements, regardless of the type of pictures presented. To do so, the scores were averaged for each participant across conditions (resulting in 1 score for each participant) and Mann-Whitney *U* tests were conducted between dogs' and children's scores for these 3 measures.

## Results

### Dogs

Within a 5-second presentation time, the dogs spent on average 3.87s±0.11 looking at the pictures, and displayed 1.64±0.08 interchanged fixations between the left and right side of the pictures. The latency of first visible eye movement was on average 1.21s±0.08 after picture onset. Regarding these 3 measures, no significant differences between image categories were found (Friedman's tests, *p*>0.05). The averaged cumulative viewing time, in seconds, directed to the ‘left’, ‘right’, ‘central’ and ‘out’ of the pictures is presented in Table 1.

**Table 1:** Dogs' viewing time.

		Gaze direction			
Image	Emotional valence	Left	Right	Central	Out
Dog face	Negative	1.60±0.20	0.57±0.09	1.95±0.17	0.88±0.16
	Neutral	1.26±0.16	0.87±0.14	1.55±0.18	1.32±0.23
	Positive	0.65±0.11	1.16±0.14	2.09±0.17	1.10±0.20
Human face	Negative	1.34±0.19	0.70±0.12	1.53±0.17	1.43±0.26
	Neutral	1.24±0.16	0.72±0.15	2.15±0.20	0.89±0.22
	Positive	1.19±0.21	0.88±0.14	2.15±0.18	0.79±0.22
Object		0.98±0.15	1.12±0.20	1.77±0.15	1.12±0.18

Mean time and standard error (mean±SE), in seconds, dogs spent looking on the ‘left’, ‘right’, ‘central’ and ‘out’ of the pictures for each image category.

#### Standard vs. mirror sessions

The analysis conducted on the 16 dogs who completed both sessions (standard and mirror) indicated no significant differences in LI (First look), or LI (Total look) between the 2 sessions for all type of images (Table 2), suggesting that the lateral eye movements in dogs were not affected by any subtle visual asymmetrical information contained in the face and object pictures.

**Table 2:** Dogs' standard and mirror sessions.

		LI (First Look)			LI (Total Look)		
		Session		P	Session		P
Image	Emotional valence	Standard	Mirror		Standard	Mirror	
Dog face	Negative	0.32±0.14	0.39±0.11	0.71	0.24±0.05	0.06±0.09	0.14
	Neutral	0.03±0.15	0.06±0.15	0.88	0.12±0.08	−0.01±0.07	0.17
	Positive	−0.16±0.19	0.11±0.13	0.13	−0.14±0.06	−0.01±0.07	0.08
Human face	Negative	−0.01±0.18	0.17±0.20	0.48	0.14±0.06	0.10±0.10	0.67
	Neutral	0.33±0.15	0.31±0.15	0.92	0.20±0.06	0.14±0.08	0.56
	Positive	0.04±0.16	−0.12±0.15	0.44	0.08±0.08	0.00±0.07	0.48
Object		−0.25±0.17	−0.03±0.15	0.34	−0.01±0.10	0.05±0.06	0.48

Dogs' scores (mean±SE) and difference (P value) between standard and mirror sessions regarding both LI (First look) (2-tailed Wilcoxon Signed Rank tests) and LI (Total Look) (2-tailed paired *t*-tests), for each image category.

### First look

**Figure 3:**
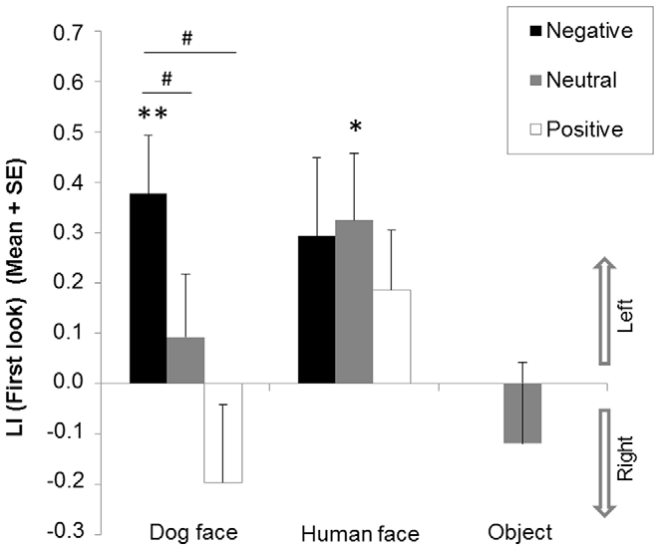
Dogs' first look. Mean Lateralisation Index (LI) and standard error (SE) of dogs' *First look* for each image category (dog faces, human faces, and objects) and each emotional valence (negative, neutral and positive). *Significant deviation from chance (0) (2-tailed Wilcoxon signed rank tests, * *p*<0.05; ** *p*<0.01). ^#^ Significant differences between valence of emotions (Wilcoxon signed rank tests, *p*<0.05).

The results are illustrated in Figure 3. There was a significant effect of the emotional valence of the pictures on the LI (First look) within dog face pictures (*χ^2^*(2) = 6.24, *p* = 0.04). Significant differences lay between negative and positive valence (*Z* = −2.26, *p* = 0.02) as well as between negative and neutral valence (*Z* = −2.15, *p* = 0.03). The difference between neutral and positive valence was not significant (*Z* = −1.53, *p* = 0.13). No effect of emotional valence was observed regarding human face pictures (*χ^2^*(2) = 2.26, *p* = 0.32).

Compared to chance, there was a significant left bias towards negative emotion (*Z* = −2.84, *p* = 0.004) but no significant bias towards neutral (*Z* = −0.92, *p* = 0.36) and positive emotions (*Z* = −1.06, *p* = 0.29) for the LI (First look) for dog pictures. Regarding human faces, there was a significant bias towards the left regarding neutral emotions (*Z* = −2.19, *p* = 0.03) and no significant bias towards positive emotions (*Z* = −0.66, *p* = 0.51). A possible non-significant trend for a left bias was observed for the negative emotion (*Z* = −1.77, *p* = 0.08). No significant variation from chance was revealed regarding object pictures (*Z* = −0.75, *p* = 0.45).

### Total look

**Figure 4:**
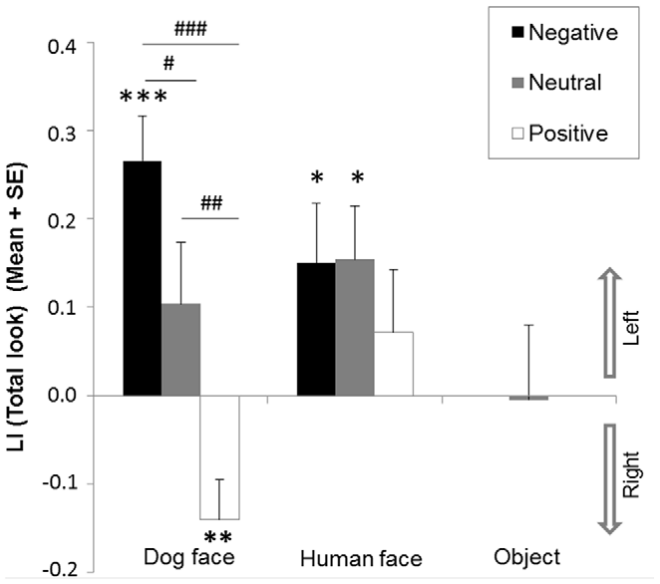
Dogs' total look. Mean Lateralisation Index (LI) and standard error (SE) of dogs' *Total look* for each image category (dog faces, human faces, objects) and each emotional valence (negative, neutral, positive) *Significant deviation from chance (0) (2-tailed 1-sample *t*-test, *p<0.05; **p<0.01; ***p<0.001). ^#^ Significant differences between valence of emotions (Post-hoc tests within ANOVA; ^#^ *p*<0.05; ^##^ *p*<0.01; ^###^ *p*<0.001).

The results are illustrated in Figure 4. There was no significant effect of face category (*F*(1,20) = 0.80, *p* = 0.38) but a main effect of emotional valence (*F*(2,40) = 8.22, *p* = 0.001) on LI (Total look). A significant interaction between face category and emotional valence was also found (*F*(2,40) = 4.44, *p* = 0.02). The effect of emotional valence was present only towards dog faces (*F*(2,40) = 18.32, *p*<0.001) and not towards human faces (*F*(2,40) = 0.49, *p* = 0.62). Regarding dog faces, post-hoc tests revealed significant differences between each emotional valence: negative and positive (*p*<0.001), neutral and positive (*p* = 0.001) as well as between negative and neutral emotional valence (*p<*0.05).

There was a significant left bias towards negative expression (*t*(20) = 5.30, *p*<0.001), no significant bias towards neutral expression (t(20) = 1.48, *p* = 0.15), and a significant right bias towards positive expressions (*t*(20) = −3.09, *p* = 0.01) on the LI (Total look) for the dog faces. Regarding human faces, a left bias was observed towards both negative (*t*(20) = 2.23, *p* = 0.04) and neutral expressions (*t*(20) = 2.53, *p* = 0.02); no significant bias was found towards positive expression (*t*(20) = 0.99, *p* = 0.33). No significant variation from chance level was revealed regarding object pictures (*t*(20) = −0.06, *p* = 0.96).

#### Children

Within a 5-second presentation time, the children spent on average 4.51s±0.08 viewing the pictures. There was a significant effect of emotional valence regarding the time spent looking at human faces (χ^2^(2) = 8.98, p = 0.01), with a significantly shorter viewing time for neutral expressions compared to negative (4.14s±0.19 vs. 4.70s±0.11; p = 0.006) or positive ones (4.14s±0.19 vs. 4.72s±0.13; p = 0.003). No such effects were observed regarding the pictures of dog faces (χ^2^(2) = 3.45, p = 0.18).

During the presentation of the pictures, the children displayed on average 3.49±0.23 interchanged fixations between the left and right side of the pictures. There was a significant effect of emotional valence on this measure for human faces (*χ^2^*(2) = 9.41, *p* = 0.01), with a greater number of left-right fixations towards negative expressions compared to neutral ones (4.03±0.34 vs. 3.50±0.36; *p* = 0.01). No such effects were found regarding the pictures of dog faces (*χ^2^*(2) = 2.00, *p* = 0.37).

The latency of the first visible eye movement was on average 0.66s±0.05 after the presentation of the pictures. No significant effect of emotional valence regarding either human or dog face pictures was found (Friedman's tests <0.05).

The averaged cumulative viewing time, in seconds, directed to the ‘left’, ‘right’, ‘central’ and ‘out’ of the pictures are presented in Table 3.

**Table 3:** Children's viewing time.

		Gaze direction			
Image	Emotional valence	Left	Right	Central	Out
Dog face	Negative	1.71±0.13	1.03±0.15	1.91±0.11	0.35±0.07
	Neutral	1.42±0.15	1.18±0.15	1.63±0.17	0.77±0.18
	Positive	1.65±0.15	1.25±0.14	1.52±0.14	0.57±0.13
Human face	Negative	1.78±0.16	1.33±0.11	1.59±0.11	0.30±0.11
	Neutral	1.54±0.14	1.30±0.20	1.30±0.13	0.86±0.19
	Positive	1.99±0.16	1.26±0.17	1.47±0.13	0.28±0.14
Object		1.49±0.18	1.37±0.16	1.82±0.12	0.32±0.09

Mean time and standard error (mean±SE), in seconds, children spent looking on the ‘left’, ‘right’, ‘central’ and ‘out’ of the pictures for each image category.

### Standard vs. mirror sessions

The analysis conducted on the 15 children who completed both sessions (standard and mirror) indicated no significant differences in LI (First look), or LI (Total look) between the 2 sessions for all type of images (Table 4). Therefore, the lateral eye movements presented by 4-year-olds children were not affected by any visual asymmetrical information contained in the pictures.

**Table 4:** Children's standard and mirror sessions.

		LI (First Look)			LI (Total Look)		
		Session		P	Session		P
Image	Emotional valence	Standard	Mirror		Standard	Mirror	
Dog face	Negative	0.51±0.14	0.61±0.13	0.32	0.19±0.05	0.19±0.06	0.99
	Neutral	0.36±0.18	0.30±0.18	0.75	0.08±0.07	0.11±0.06	0.76
	Positive	0.51±0.15	0.61±0.15	0.68	0.11±0.07	0.10±0.06	0.90
Human face	Negative	0.51±0.15	0.59±0.15	0.62	0.14±0.05	0.16±0.04	0.64
	Neutral	0.57±0.10	0.46±0.18	0.55	0.08±0.06	0.16±0.06	0.12
	Positive	0.54±0.15	0.50±0.20	0.90	0.21±0.05	0.18±0.06	0.66
Object		−0.13±0.17	0.17±0.15	0.07	0.08±0.06	0.16±0.06	0.12

Children' scores (mean±SE) and difference (P value) between standard and mirror sessions regarding both LI (First look) (2-tailed Wilcoxon Signed Rank tests) and LI (Total Look) (2-tailed paired *t*-tests), for each image category.

### First look

**Figure 5:**
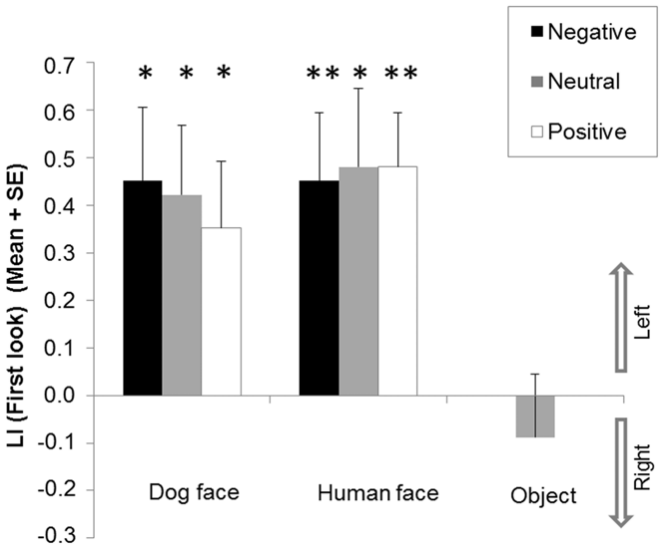
Children' first look. Mean Lateralisation Index (LI) and standard error (SE) of children's *First look* for each image category (dog faces, human faces, and objects) and each emotional valence (negative, neutral, positive). *Significant deviation from chance (0) (2-tailed Wilcoxon signed rank tests, * *p*<0.05; ** *p*<0.01).

The results are illustrated in Figure 5. There was no significant effect of emotional valence of the pictures on the LI (First look) within dog pictures and within human pictures (dog faces: χ2(2) = 0.52, p = 0.77; human faces: χ2(2) = 0.04, p = 0.98).

Compared to chance, there was a significant left bias towards all face pictures on the LI (First look), regardless of the facial expression presented (all *p*<0.02). No significant variation from chance level was revealed for object pictures (*p* = 0.55).

### Total look

**Figure 6:**
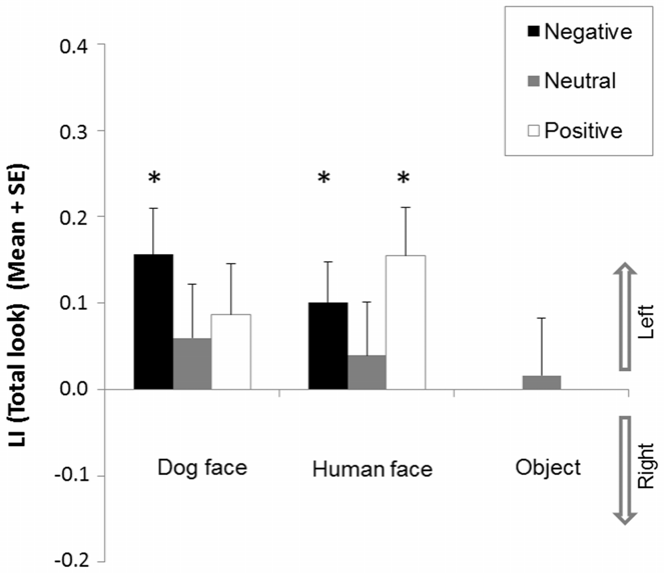
Children's total look. Mean Lateralisation Index (LI) and standard error (SE) of children's *Total look* for each image category (dog faces, human faces, and objects) and each emotional valence (negative, neutral, positive). *Significant deviation from chance (0) (2-tailed 1-sample *t*-test, *p<0.05).

The results are illustrated in Figure 6. There were no significant main effects of face category (*F*(1,16) = 0.003, *p* = 0.95) or emotional valence (*F*(2,32) = 2.45, *p* = 0.10) nor was any interaction between these 2 factors (*F*(2,40) = 1.28, *p* = 0.29) on the LI (Total look).

There was a significant left bias towards negative expression (*p* = 0.01) and no significant bias towards neutral (*p* = 0.35) or positive expressions (*p* = 0.16) on the LI (Total look) for the dog faces. Regarding the human faces, a left bias was observed towards both negative (*p* = 0.048) and positive expressions (*p* = 0.02) and no significant bias towards neutral expression was found (*p* = 0.54). No significant variation from chance level was revealed regarding object pictures (*p* = 0.81).

### Dogs & Children

Overall there were large differences between participant groups on the total amount of time spent looking at the images, the amount of interchanged fixations between the left and right side of the pictures and the latency of first detectable eye movements between dogs and children. Specifically, dogs spent less time looking at the pictures (3.87s±0.11 vs. 4.51s±0.08; *p*<0.001), showed less left-right interchanged fixations (1.64±0.08 vs. 3.49±0.23; *p*<0.001) and presented a larger latency of first eye movement (1.21s±0.08 vs. 0.66s±0.05; *p*<0.001).

### Total look

**Figure 7:**
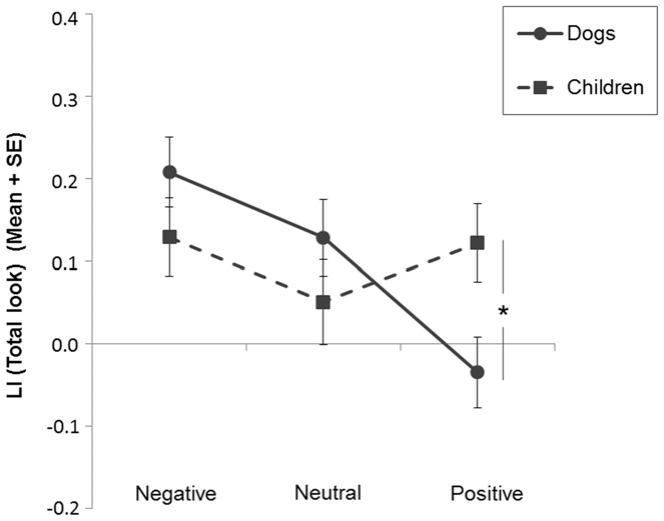
Dogs' and children's total look. Mean Lateralisation Index (LI) and standard error (SE) of both dogs and children's *Total look* regarding each emotional valence (negative, neutral and positive). *Significant differences between variables (ANOVA; * *p*<0.05).

The results are illustrated in Figure 7. There was a significant interaction between the type of participant and the emotional valence (*F*(2,72) = 6.30, *p* = 0.003) on the LI (Total look). This interaction was present only regarding positive emotions (*F*(1,36) = 6.09, *p* = 0.02), dogs presenting significantly lower scores compared to children. No such differences were observed between dogs and children regarding negative (*F*(1,36) = 1.53, *p* = 0.22) or neutral emotions (*F*(1,36) = 0.34, *p* = 0.57).

## Discussion

To our knowledge, the present study is the first of its kind demonstrating differential brain lateralisation in processing facial expressions associated with distinct emotional valence in a non-human species. We observed clear differential processing of conspecific facial expressions in dogs depending on their emotional valence, with no gaze laterality towards neutral conspecific expressions but a left gaze bias while looking at negative facial expressions and a right gaze bias towards positive ones. Similar observations regarding lateralisation of tail-wagging in dogs were made when exposed to negative and positive stimuli [39]. Regarding cerebral lateralisation, these observations are consistent with the *Valence Model*, with the right hemisphere mainly involved in the processing of negative emotions and the left hemisphere mainly involved in the processing of positive emotions. The development of such facial emotion processing skills in domestic dogs within an intraspecific context could play a major role in coordinating social interactions and facilitate group cohesion, as suggested regarding primate species [56]. The question whether these abilities in domestic dogs are inherited from their wolf ancestors living in highly complex societies still has to be determined.

Regarding human faces, dogs presented less clear variations in gaze laterality compared to conspecific faces. However, the results indicated a differentiation between non-positive (negative and neutral) and positive expressions, as a left gaze bias was observed towards both negative and neutral expressions but not towards positive expressions (no bias). One interpretation of these results might be that domestic dogs are not sensitive to negative human facial expressions and therefore process these in a similar way to neutral faces. However, the results can also be interpreted as neutral facial expressions being processed as potentially negative, given their lack of clear approach signals. Indeed, studies assessing the judgment of human faces by human participants found that prototypical neutral faces (being relaxed, presenting no facial muscle contraction, [57]) are not evaluated as neutral but in a negative way such as appearing cold or threatening, possibly due to the social convention to signal approval in normal interactions [58]. Because of this, some authors now use morphed faces (75% neutral, 25% happy) as a new ‘neutral’ baseline [59]. This possibility would also lead to reconsideration of the cerebral mechanisms underlying the left gaze bias originally found towards the neutral human face (but not towards neutral dog or monkey faces) in dogs [40] and replicated here: i.e. is the right hemispherical dominance associated with face and/or negative emotion processing? Further research is therefore warranted in this area.

In order to estimate the type of strategy domestic dogs use to process facial expressions of emotion in comparison to that of humans, dogs' lateralisation responses were directly compared to that of 4-year-old children within a similar paradigm. This comparative aspect allowed us to establish some major differences between these two groups. Firstly, dogs and 4-year-olds presented a discrepancy regarding the consistency of the type of measure used to assess laterality of eye movements. *First look* and *Total look* measures showed a qualitatively similar result pattern in dogs but a different pattern in 4-year-olds. Children tended to look first on the left side of the pictures for all types of faces (human and dog) and for all types of facial expressions, but left biases regarding the amount of time spent looking at one or other side of the pictures were observed only for certain facial expressions. The pattern expressed by 4-year-olds fit the idea that initial fixation could correspond to a reflexive response to the processing of gist facial configuration whereas overall fixation could be more associated with the processing of some specific type of facial information, such as emotions. This hypothesis is consistent with other studies in adult humans that have found a leftward eye movement bias when analyzing the initial saccade but a less clear result when looking at the overall fixation durations [41], [60], suggesting that initial and total fixations could be governed by different cognitive mechanisms. Regarding dogs, no such evidence could be found as both measures revealed a similar type of bias. Could initial and overall fixations in dogs be driven by different cognitive mechanisms to that of 4-year-olds? Some large differences between dogs and 4-year-olds in general scanning behaviour and attention towards the stimuli presented make this question difficult to answer. Indeed, besides dogs spending less time looking at the pictures compared to 4-year-olds (4.8s vs. 4.5s respectively), they also displayed less than half of the amount of left-right interchanged fixations towards the pictures (1.6 vs. 3.5) and showed twice as high a latency to first eye movement (1.2s vs. 0.6s). Such a low number of interchanged left-right fixations towards the pictures means the overall fixation is heavily dependent upon the initial one. Considering that dogs' general scanning behaviour could result in a confounding effect between both measures, some potential differences in laterality of eye movement regarding the *First Look* and the *Total Look* measure could not have been expected in this population.

A second notable difference between dogs and 4-year-olds is the type of lateralisation elicited by processing facial expressions depending on their emotional valence. While the lateralised responses observed in domestic dogs strongly support the *Valence Model*, the results from 4-year-olds fit the *Right Hemisphere Model*. Indeed, both negative and positive facial expressions displayed by human faces elicited a left gaze bias in 4-year-olds while neutral expressions did not. As to dog faces, a left bias could also be observed regarding negative expressions. No bias regarding either neutral or positive expressions was found. In this case, the absence of bias regarding dog positive expressions could be explained by a lack of exposure to dogs by most of the tested children (only 3 children in the present sample were regularly exposed to dogs, that is, had a dog at home). Nevertheless, being sensitive to threating facial expressions would provide a clear advantage in terms of harm avoidance and might develop with less/no exposure to the specific stimulus. These questions warrant further research.

When both dogs' and children's responses were directly compared, a clear difference regarding the processing of positive facial expressions was observed. Indeed, dogs and children presented opposite responses regarding positive facial expressions, a lateralization to either the right or left of the pictures (respectively), notably regarding conspecific faces. In the literature, both the *Right Hemisphere* and *Valence* models have received strong empirical support [35]. However, the various types of methods and stimuli employed between studies could be at the origin of this difference. The present study provides evidence of a specific use of one or the other model within a similar experimental paradigm, regarding dogs and 4-year-old humans. Therefore, the engagement of one or the other brain hemisphere for the processing of emotional functions could be species-dependent. Nevertheless, it cannot be excluded that lateralization of emotional processing could be subject to developmental changes. Indeed, previous studies have highlighted refinements in degree of lateralization for some functions (e.g. handedness in humans [61]) and even shift in bias for the use of one or the other hemisphere (e.g. visual recognition of conspecifics in domestic chicks [62]) through development. Thus, the observed 4-year-olds' viewing strategy towards facial expression pictures in this study might not be the one used by human adults.

In the present study the visual stimuli included posed (acted) human facial expressions and evoked (genuine) dog facial expressions. Posed and evoked facial expressions might induce different responses in observers, the first one tending to be more extreme in intensity but the second one being more natural. Indeed, different processing of one or the other type of facial expressions has been found in human adults [63], [64]. Given this consideration, it could be argued that the difference in our stimuli may have contributed to distinct processing of human and dog facial expressions. However, people commonly exaggerate their facial expressions when interacting with children [65], and a similar phenomenon could also be found when people interact with pet dogs. Indeed, similarity in communicative behaviours towards both children and dogs has already been highlighted [66]. We can therefore wonder what is a ‘natural’ human facial expression in a child or a dog's mind? Further research is warranted in this direction.

To conclude, this study provides clear evidence of sensitivity to conspecific facial expressions in domestic dogs and also, to a lesser extent, to human facial expressions. Dogs presented differential lateralised eye movements when processing pictures of each species face, depending on the emotional valence of the facial expressions viewed. Additionally, the comparative study with 4-year-old children highlighted a different type of lateralisation regarding the type of emotion processed.
